# An Evaluation of the Corrosion Inhibition Performance of Chitosan Modified by Quaternary Ammonium Salt for Carbon Steel in Stone Processing Wastewater

**DOI:** 10.3390/molecules29143401

**Published:** 2024-07-19

**Authors:** Jingjing Xiang, Chaofan Mo, Chao Peng, Lin Yang, Tingtao Wan, Yuntian Song, Xuanhui Lei, Pu Liu, Bo Gao, Dajun Ren, Chong Zhao, Yanjun Huang, Yi Wang, Lei Zhang

**Affiliations:** 1 School of Chemistry and Environmental Engineering, Wuhan Polytechnic University, Wuhan 430023, China; jingjingxiang605@163.com (J.X.); chaofanmo@foxmail.com (C.M.); chaochem@foxmail.com (C.P.); 13980921145@163.com (T.W.); pt1690739250@163.com (Y.S.); leixh2790002706@163.com (X.L.); zhaochong426@126.com (C.Z.); hyj.321@163.com (Y.H.); wangyi2020@whpu.edu.cn (Y.W.); 2 R&D Center of Wuhan Iron and Steel Company, Wuhan 430080, China; e83082@baosteel.com; 3 Wuhan Huadet Environmental Protection Engineering & Technology Co., Ltd., Wuhan 430080, China; 18627808373@163.com; 4 School of Resources and Environmental Engineering, Wuhan University of Science and Technology, Wuhan 430080, China; dj_ren@163.com

**Keywords:** corrosion inhibitor, QWSC, stone processing wastewater, electrochemistry, corrosion mechanism

## Abstract

Chitosan was used as the raw material. A quaternization reaction was carried out between 2,3-epoxypropyltrimethylammonium chloride and water-soluble chitosan to prepare quaternary ammonium salt water-soluble chitosan (QWSC), and its corrosion inhibition performance against the corrosion of carbon steel in stone processing wastewater was evaluated. The corrosion inhibition efficiencies of QWSC on carbon steel in stone processing wastewater were investigated through weight loss, as well as electrochemical and surface morphology characterization techniques. The results show that QWSC has superior corrosion inhibition performance for A3 carbon steel. When an amount of 60 mL·L^−1^ is added, the corrosion inhibition efficiency can reach 59.51%. Electrochemical research has shown that a QWSC inhibitor is a mixed-type corrosion inhibitor. The inhibition mechanisms of the QWSC inhibitor revealed that the positive charge on the surface of carbon steel in stone wastewater was conducive to the adsorption of Cl^−^ in the medium, which produced an excessive negative charge on the metal’s surface. At the same time, the quaternary ammonium cation and amino cation formed in QWSC in stone processing wastewater can be physically absorbed on the surface of A3 carbon steel, forming a thin-film inhibitor to prevent metal corrosion.

## 1. Introduction

Using a corrosion inhibitor is an effective method to prevent the corrosion of metal materials. Inorganic corrosion inhibitors have been gradually banned due to their large dosage and high toxicity. On the other hand, green and highly efficient natural macromolecular organic corrosion inhibitors have the advantages of being renewable, non-toxic, and biodegradable, and they have attracted much attention. Most organic inhibitors contain heteroatoms like N, O, S, and P, with the most effective inhibitors often being compounds containing π bonds [1]. These atoms are able to react with active sites on the surface of carbon steel and display excellent anti-corrosion effects [2]. Chitosan (CS), as an effective metal substrate inhibitor, has the advantages of low toxicity, abundance, environmental friendliness, affordability, and biodegradability, making it a promising corrosion inhibitor [1,3]. CS is protonated in dilute acidic media and exhibits polyelectrolyte-like behavior, giving it a good membrane-forming capacity and adhesivity to metal surfaces, thus allowing it to form a protective barrier. Moreover, the corrosion resistance of CS is mainly due to OH (hydroxyl) and NH_2_ (amino), along with solitary electron pairs, being able to coordinate with the metal surface. However, CS itself has low solubility and low surface adhesion, limiting its application in the field of corrosion inhibitors. Therefore, researchers have modified CS to maximize its application potential in this field [4,5,6,7]. Quaternary ammonium-modified chitosan not only increases its solubility but also improves its corrosion inhibition, which has become a popular research topic regarding modified chitosan [8].

Previously, we synthesized a series of organic and inorganic corrosion inhibitors and achieved superior results in inhibiting carbon steel corrosion in stone processing wastewater [9,10]. Herein, a quaternary ammonium salt water-soluble chitosan (QWSC) inhibitor was synthesized by modifying chitosan. The corrosion inhibition performance of QWSC on carbon steel in stone processing wastewater was investigated systematically via weight loss measurements, scanning electron microscopy (SEM), atomic force microscopy (AFM), and electrochemical methods. The adsorption type of the QWSC inhibitor on the steel specimen surface was analyzed using XPS; further, the inhibition mechanism was also described.

## 2. Results

### 2.1. Measurement of Weight Loss

**Figure 1:**
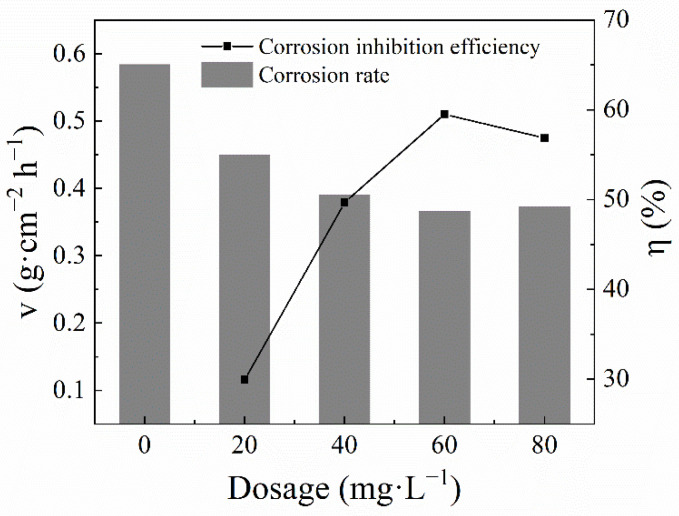
Effect of composite inhibitor concentrations on inhibition effect.

The effect of adding different concentrations of the QWSC inhibitor on a corrosive material in stone processing wastewater was measured as shown in Figure 1. As the QWSC concentration increased, the corrosion inhibition efficiency increased and then decreased. A maximum inhibition efficiency of 59.51% was obtained for the QWSC inhibitor at 60 mg·L^−1^.

### 2.2. Electrochemical Measurements

#### 2.2.1. Open-Circuit Potential

**Figure 2:**
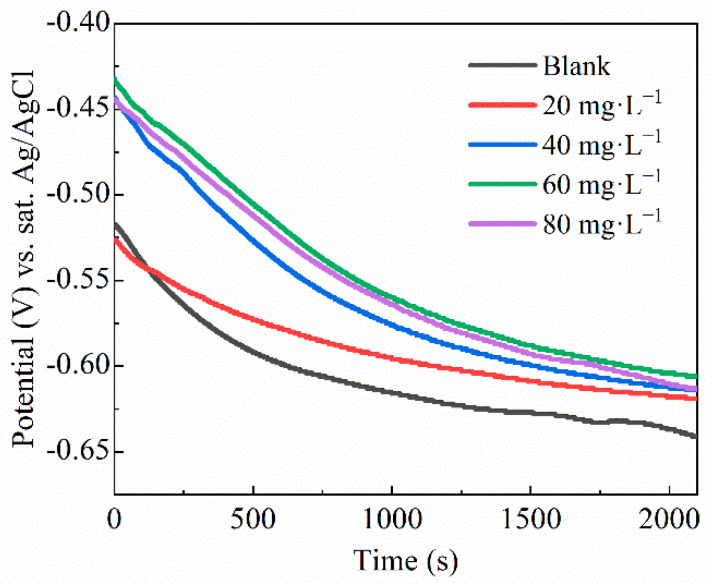
OCP evolution vs. time of the A3 carbon steel in stone processing wastewater containing different concentrations of QWSC inhibitor.

The alteration in the OCP, that is, the potential generated on a working electrode (A3 carbon steel) in stone processing wastewater versus Ag/AgCl potential, with a time of (2100 s) for the composite inhibitor, is shown in Figure 2. Generally, the curves of all the examined samples have almost similar features. It can be seen that increasing the amount of the QWSC inhibitor promotes the passivation of the carbon steel electrode in stone processing wastewater. The OCP of the samples decreased significantly in the uninhibited solution. In the presence of the QWSC inhibitor, the OCP moved in the positive direction, indicating that the QWSC inhibitor was more inclined to inhibit the corrosion reaction at the anode.

#### 2.2.2. Potentiodynamic Polarization Tests

**Figure 3:**
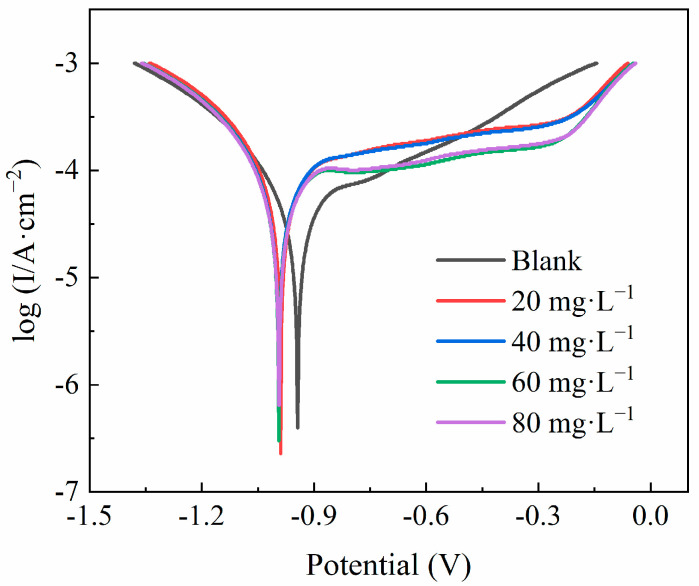
Polarization curves of A3 carbon steel in stone processing wastewater with different concentrations of QWSC inhibitor.

The polarization curves and electrochemical parameters for A3 carbon steel electrodes at different concentrations of the QWSC inhibitor in stone processing wastewater are shown in Figure 3. As shown in Figure 3, comparing the polarization curves in stone processing wastewater, the corrosion current densities corresponding to the cathodic and anodic reaction decrease with the addition of the QWSC inhibitor, and also, the self-corrosion potentials of the Tafel polarization curves shifted in the direction of negative potentials, indicating the relatively high inhibition efficiency.

The degree of surface coverage (θ) was calculated using the following Equation (1) [11,12]:

(1) $$\theta =1-\frac{i_{corr}}{i_{corr}^{0}}$$

where $i_{corr}$ and $i_{corr}^{0}$ are the corrosion current density for the specimens in corrosive media with and without the QWSC inhibitor. The corresponding values of the corrosion potential ($E_{corr}$), corrosion current density ($i_{corr}$), and the degree of surface coverage θ are listed in Table 1. θ is used to indicate the passivation degree of the QWSC inhibitor on metals [11]. The θ values were gradually enhanced and reduced with the increasing concentrations of the QWSC inhibitor, which were consistent with the inhibition efficiencies (See Figure 1). Furthermore, the corrosion potential ($E_{corr}$) moved about 45~50 mV in the cathodic potential direction after the addition of the QWSC inhibitor—as shown in Table 1—and the difference was less than ±85 mV, indicating that QWSC was a mixed-type corrosion inhibitor with predominantly cathodic action [13,14].

#### 2.2.3. Electrochemical Impedance

**Figure 4:**
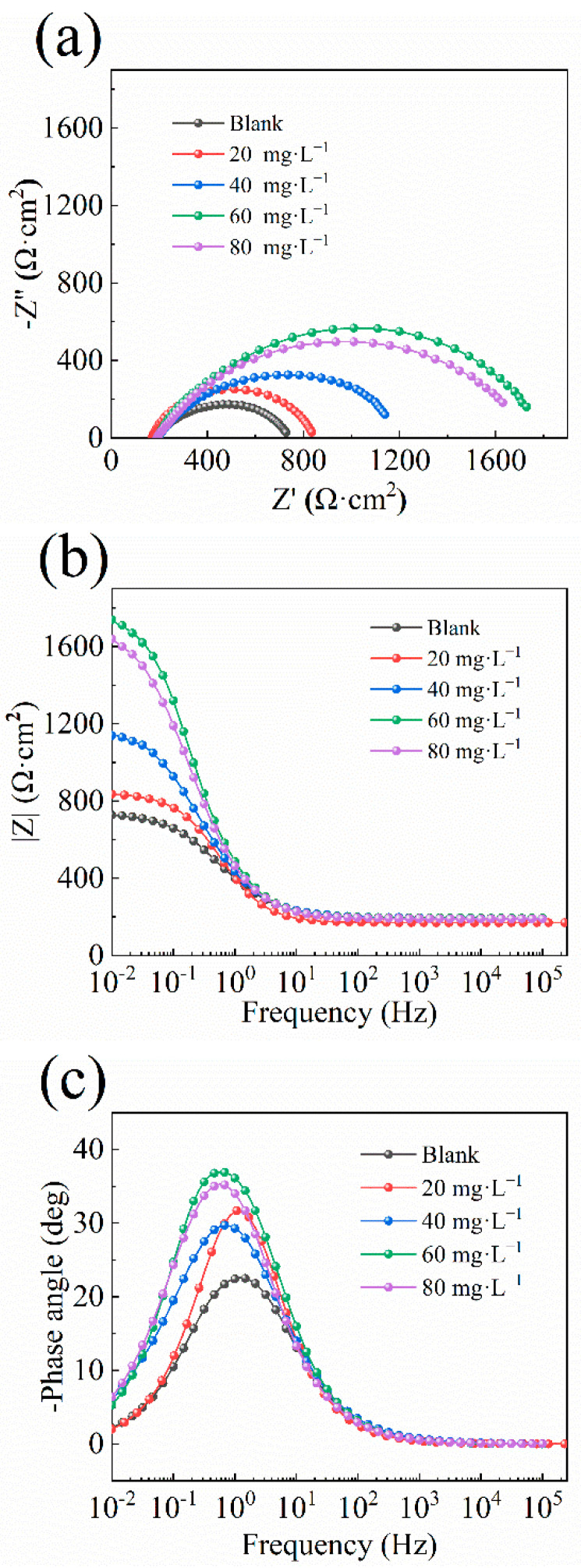
EIS plots: (**a**) Nyquist plots; (**b**) Bode impedance modulus plots; (**c**) Bode phase plots for A3 carbon steel in stone processing wastewater containing different concentrations of QWSC inhibitor.

Figure 4 shows the electrochemical impedance spectra of A3 carbon steel in stone processing wastewater containing different concentrations of the QWSC inhibitor. Figure 4a–c correspond to the Nyquist plot, Bode impedance modulus plot, and Bode phase angle plot, respectively. Generally speaking, the larger diameters of Nyquist semi-circles are attributes of the higher corrosion resistance systems of inhibitors compared to the blank solution [15]. As shown in Figure 4a, the size of the diameters of the impedance arc with the addition of the corrosion inhibitor was larger compared to the curve without the addition of QWSC, indicating the better inhibition performance of the QWSC inhibitor. The best corrosion inhibition efficiency was obtained at the QWSC concentration of 60 mg·L^−1^, which was consistent with the polarization curve results. In Figure 4b, it can be seen that |Z|_0.01Hz_ showed a tendency to increase and then decrease with the increase in QWSC concentration. The value of |Z|_0.01Hz_ was at its maximum when the concentration of QWSC was 60 mg·L^−1^. The phase angle plots had the same trends as shown in Figure 4c.

**Figure 5:**
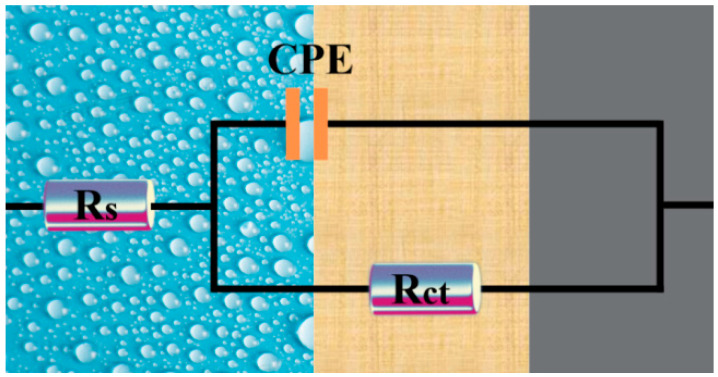
EIS equivalent circuit.

Figure 5 shows the EIS equivalent circuit diagram of A3 carbon steel in stone processing wastewater containing different concentrations of the QWSC inhibitor, and the results of the EIS curve parameter fitting are shown in Table 2. In Figure 5, Rs is the solution resistance; CPE is the capacitance of the double electric layer at the interface between carbon steel and solution; and R_ct_ is the charge-transfer resistance [12]. The corrosion inhibition efficiency ($IE_{EIS}$) was calculated using the following Equation (2) [15]:

(2) $$IE_{EIS}\%={(R}_{\mathrm{ct}}-R_{\mathrm{ct}}^{0}{)/R}_{\mathrm{ct}}\times 100$$

where R_ct_ and R^0^_ct_ are the charge-transfer resistance for the specimens in corrosive media with and without the QWSC inhibitor. The values of R_ct_ can be used to evaluate the electrochemical corrosion rate, the lower the value of R_ct_, the faster the corrosion reaction rates [16,17]. As shown in Table 2, it can be seen that the R_ct_ value reached its maximum when the QWSC inhibitor concentration was 60 mg·L^−1^, showing superior protection for the A3 carbon steel. Increased R_ct_ values with the QWSC inhibitor concentration were based on the increase in inhibitor surface coverage, which led to an increase in inhibitor efficiency. The observed increment in the values of the R_ct_ at 60 mg·L^−1^ could be due to the formation of a precipitate of corrosion products as well as a Fe-inhibitor-type complex that further protected the metal surface. From the fitting parameters in Table 2, the corrosion inhibition efficiency was 59.15% when the QWSC concentration reached 60 mg·L^−1^.

### 2.3. Surface Topography Analysis

#### 2.3.1. SEM Measurement

**Figure 6:**
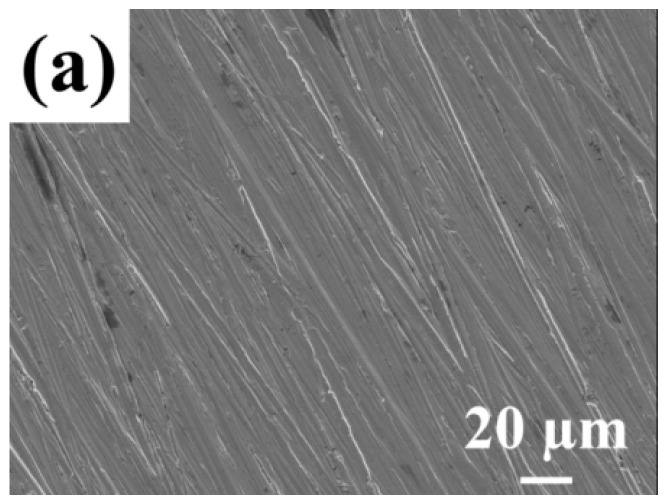

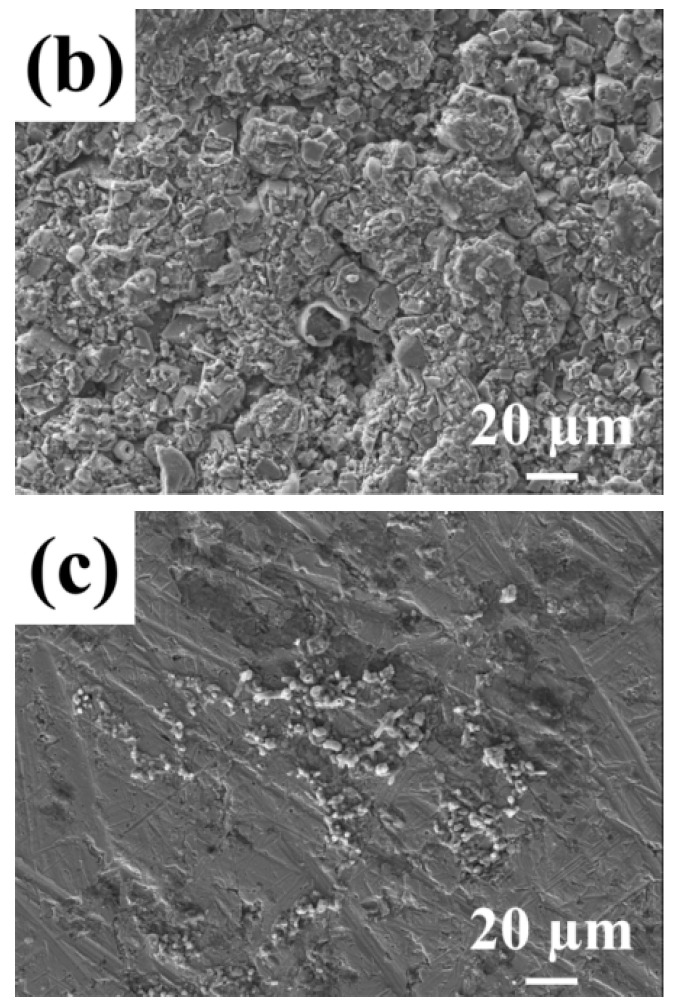
Scanning electron microscopy micrograph of A3 carbon steel: (**a**) polished A3 carbon steel sample; (**b**) after immersion in stone processing wastewater for 7 d; (**c**) after immersion in stone processing wastewater with 60 mg·L^−1^ QWSC inhibitor for 7 d.

Scanning electron microscopy was utilized to observe the morphology of A3 carbon steel before and after corrosion. As shown in Figure 6, the SEM images presented depict (a) polished metal samples; (b) blank samples (without the added corrosion inhibitor and immersed for 7 days); and (c) A3 carbon steel samples immersed for 7 days in the presence of 60 mg·L^−1^ of the QWSC inhibitor, respectively. The observation in Figure 6a shows a flat surface with a deep and clear grain, whereas Figure 6b displays that the steel surface is highly corroded and reveals a mountain-like appearance [12]. Figure 6c shows the surface covered with a thick layer of corrosive material and unevenness when 60 mg·L^−1^ of the QWSC inhibitor was added, which could be due to the localized corrosion caused by the incomplete coverage of QWSC molecules on the carbon steel surface.

#### 2.3.2. AFM Analysis

**Figure 7:**
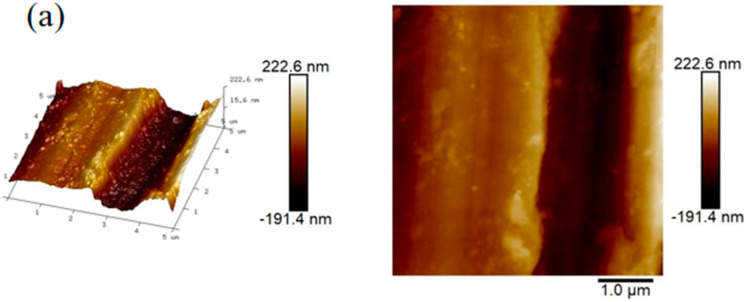

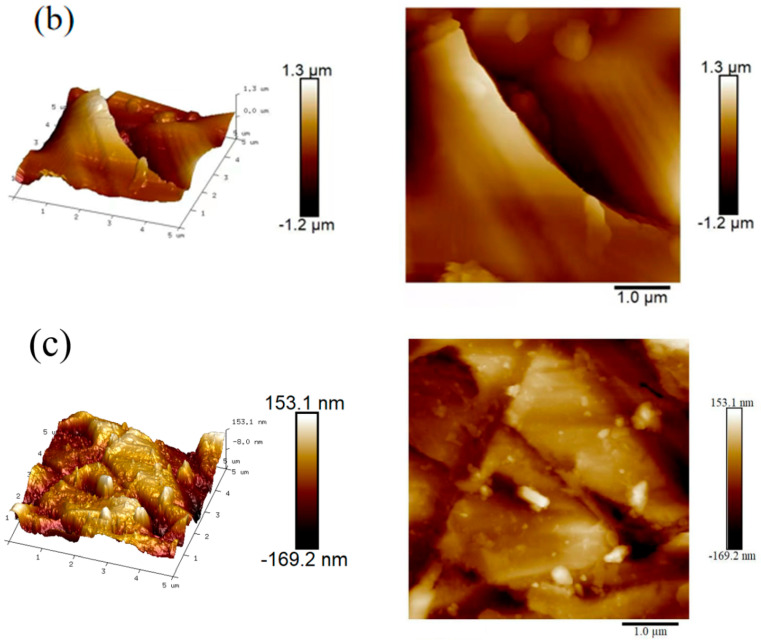
Atomic force microscopy images for (**a**) polished A3 carbon steel, (**b**) blank sample, and (**c**) A3 carbon steel immersed for 7 d in the presence of QWSC inhibitor at 60 mg L^−1^.

To further illustrate the changes in the surface of carbon steel after the addition of the QWSC inhibitor, atomic force microscopy was utilized to observe the morphology of the carbon steel surface and the obtained images are shown in Figure 7. As can be seen from Figure 7a, after polishing, the maximum height difference in the sample surface was 414 nm. Figure 7b shows the carbon steel was seriously corroded in stone processing wastewater for 7 days; the maximum height difference in the surface was 2.5 µm. After corrosion inhibitors are added, it can be seen that the surface roughness of Figure 7c is significantly lower than that of Figure 7b, and the surface height difference also decreased from 2.5 µm to 322.3 nm, indicating that the QWSC inhibitor can be used to protect the A3 carbon steel surface.

#### 2.3.3. Contact Angle Measurement

**Figure 8:**
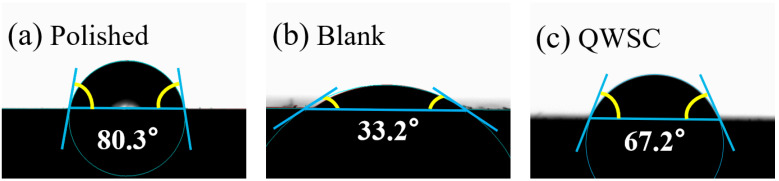
Contact angles of A3 carbon steel surfaces: (**a**) polished metal sample; (**b**) blank sample; and (**c**) A3 carbon steel sample immersed for 7 d in the presence of QWSC inhibitor at 60 mg L^−1^.

The contact angles presented in Figure 8 are (a) polished A3 carbon steel samples, (b) A3 carbon steel after 7 days of immersion in stone processing wastewater without the corrosion inhibitor, and (c) A3 carbon steel immersed in stone processing wastewater containing 60 mg L^−1^ of the QWSC corrosion inhibitor for 7 days, respectively. The stronger the hydrophobicity that the formed films have, the better their protective ability. For the blank sample, the contact angle of A3 carbon steel in stone processing wastewater after polishing was 33.2°. After adding 60 mg L^−1^ of the QWSC inhibitor, the contact angle increased to 67.2°, indicating that the corrosion inhibitor formed an adsorption film on the surface of carbon steel, which enhanced the hydrophobicity of the carbon steel surface, thus producing a corrosion inhibition effect.

### 2.4. Inhibition Mechanism

#### 2.4.1. XPS Analysis

**Figure 9:**
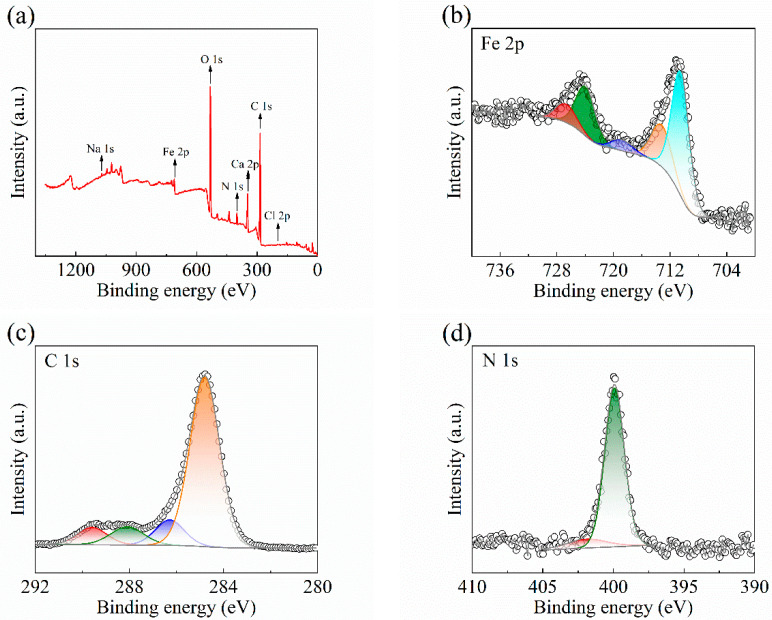

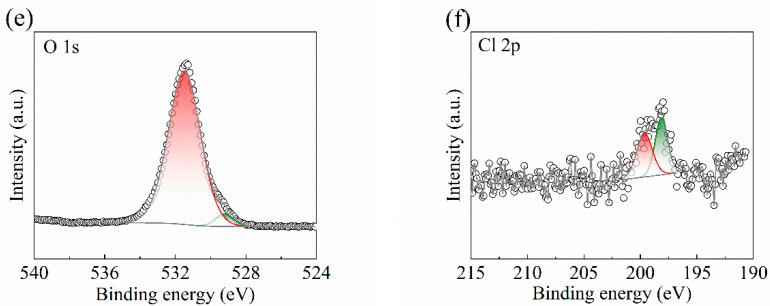
XPS survey spectrum of A3 carbon steel surface after immersion in stone processing wastewater with 60 mg·L^−1^ QWSC inhibitor for 7 days: (**a**) full XPS spectra; (**b**) Fe 2p, (**c**) C 1s, (**d**) N 1s, (**e**) O 1s; and (**f**) Cl 2p.

A3 carbon steel after corrosion in stone processing wastewater containing 60 mg L^−1^ of the QWSC inhibitor for 7 days was selected for XPS testing. The results are shown in Figure 9. The peaks of Na 1s, Fe 2p, C 1s, N 1s, O 1s, Ca 2p, and Cl 2p are shown in Figure 9a. The appearance of the O 1s peak was caused by the corrosion of oxygen and the peaks of Na 1s, C 1s, and N 1s provided evidence for the adsorption of the investigated inhibitors on the steel surface [7]. The appearance of Ca 2p peaks indicated that the corrosion inhibitor molecules were attractive to Ca^2+^ in the stone processing wastewater and these corrosion inhibitor molecules were also adsorbed onto the carbon steel surface [18]. Figure 9b presents the Fe 2p curve obtained from a steel surface that was immersed in the QWSC inhibitor solution. The peaks at binding energies 710.7 eV, 713.5 eV, 719.1 eV, 724.3 eV, and 726.9 eV were attributed to Fe_2_O_3_, FeCl_2_, FeO, FeOOH, and Fe(H_2_O) [2,12,19]. There are four chief peaks in the spectrum of C 1s in Figure 9c, which can be ascribed to C-H/C-C (284.8 eV), C-O/C-N (286.3 eV), O-C-O (288.1 eV), and O-C=O (289.6 eV), respectively [2,20]. Figure 9d shows the deconvoluted N 1s spectra at 399.9 eV and 401.9 eV, which could be assigned to C-N and protonated N atoms (-N^+^), respectively [21]. Due to the lack of a pair of electrons on the N atom, the QWSC inhibitors cannot form chemical bonds between quaternary ammonium compounds and steel surfaces. The adsorption between the corrosion inhibitor and the carbon steel surface was formed through the electrostatic gravitational force between the N atoms in the corrosion inhibitor and the molecules with different orientations on the carbon steel surface [20,22]. Figure 9e shows that the two peaks in the O 1s spectrum located at 529.2 eV and 531.5 eV are attributable to FeOOH and Fe-O, respectively [2,23,24]. There are two absorption peaks in the Cl 2p spectrum in Figure 9f, which are Cl 2p_3/2_ (198.2 eV) and Cl 2p_1/2_ (199.7 eV), which may be assigned to FeCl_2_ and FeCl_3_ produced via the corrosion reaction of the carbon steel in the stone processing wastewater [25,26].

#### 2.4.2. Quantum Chemical Computing

**Figure 10:**
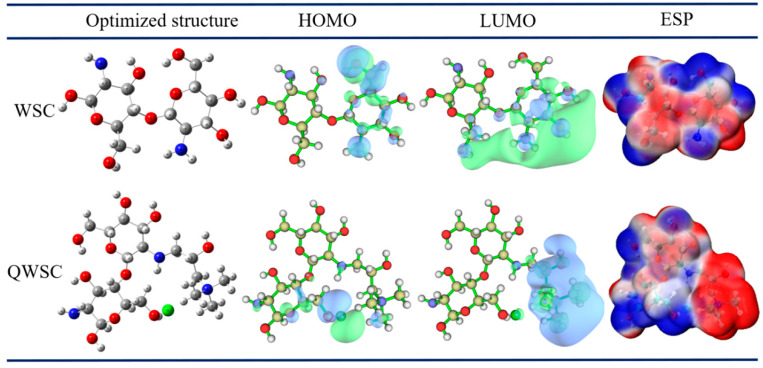
The optimized structure, HOMO, and LUMO of WSC and QWSC.

Quantum calculations were performed to study the relationship between molecular structure and inhibitory properties [27]. Figure 10 shows the geometrically optimized structures of WSC and QWSC molecules and their HOMO–LUMO distribution. The arrangement of the HOMO and LUMO orbitals reveals the high reactivity of these molecules with the metal surface [25]. An electrostatic potential (ESP) map can directly reveal the distribution of the electrostatic potential of the inhibitor molecule in space and help to quickly locate the position of the lone electron pair, i.e., potential reaction sites [28,29]. In the ESP map, the minimum point (blue sphere) represents the most active reaction sites, and the lone electron pair (red area) is attributed to the O and N atoms’ unoccupied electron orbitals. Table 3 shows the quantum chemical parameters obtained from the calculations. As shown in Figure 10, it can be found that the HOMO of WSC was mainly distributed on the hydroxymethyl and amino groups of the second ring; and the LUMO was mainly distributed at the amino group of the first ring, the amino group of the second ring, and the hydroxyl group; the HOMO of QWSC was mainly distributed on chloride ion and its surrounding N and O atoms; and the HOMO was mainly distributed on the grafted quaternary ammonium salt.

Table 3 provides a list of several quantum chemical parameters for the WSC and QWSC molecules. The computed gap energies ΔE were used to predict the adsorption abilities of the WSC and QWSC molecules on the carbon steel surface. The ΔE value of WSC was greater than that of QWSC, indicating that the adsorption of QWSC on the surface of carbon steel is greater than that of WSC. The ΔN values of WSC and QWSC were calculated to be less than 3.6, which indicated that both were able to provide electrons to the metal surface [27]. The electronegativity (χ) and transferred electrons (ΔN) obtained from quantum chemical calculations suggested that QWSC exhibited higher inhibition performance [2].

## 3. Discussion

**Figure 11:**
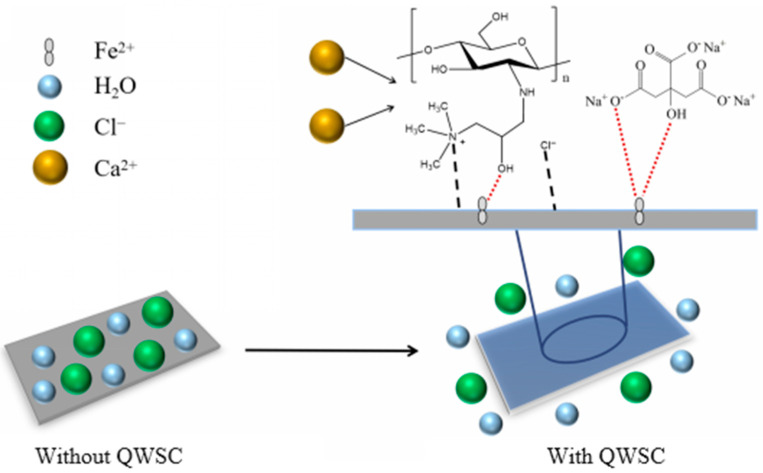
Schematic illustration of the inhibition mechanisms of QWSC inhibitor in stone processing wastewater. (Left) without QWSC inhibitor; (Right) with QWSC inhibitor.

In general, the mechanism of action of corrosion inhibitors on metal surfaces in Cl^−^ solutions may be influenced by the chemical structure of the inhibitor molecule, the nature of the metal, and the charge [30]. The high content of chloride ions in stone processing wastewater destroyed the passivation film and promoted anodic reactions. At the same time, pitting corrosion tended to occur where the passivation film was destabilized. On the basis of the above analysis, the possible corrosion inhibition mechanism of the QWSC inhibitor was proposed. Figure 11 shows the schematic illustration of the inhibition mechanism of the QWSC inhibitor in stone processing wastewater. The inhibition approach of the QWSC inhibitor could be explained as the following process. First, the aggressive chloride ions in stone processing wastewater were adsorbed on carbon steel surfaces, thereby accelerating the dissolution of Fe^2+^ ions on the surface of carbon steel. Then, the quaternary ammonium cations and amino positive ions formed by the QWSC in stone processing wastewater migrated to the surface of the carbon steel covering a large amount of Cl^−^ on the surface of A3carbon steel, and QWSC molecules could also be attached to carbon steel surface via chemisorption through the adsorption center and the formation of coordination bonds with the empty orbitals of the Fe atoms of carbon steel. Eventually, a protective layer was formed, which not only effectively isolated direct contact from the corrosive medium to the metal surface but also inhibited the cathode and anode electrochemical reactions [31,32].

## 4. Materials and Methods

### 4.1. Materials

Commercial A3 carbon steels with dimensions of 50 mm × 25 mm × 2 mm were used as the corrosive materials. The chemical compositions (wt%) of the corrosive materials are C (0.24), Si (0.12), Mn (0.64), S (0.012), and P (0.16), and the balance is Fe. Before testing, corrosive material specimens were polished and cleaned. This was finished by burnishing them sequentially using silicon carbide paper with sand particles of 150, 220, 400, 800, and 1500 and washing them with ethanol and deionized water.

The following chemicals were used for the preparation of the QWSC inhibitor: chitosan (deacetylation degree ≥ 95%, viscosity 100–200 mPa.s) and glycidyl trimethyl ammonium chloride (C_6_H_14_ClNO, >95%) were purchased from Shanghai Aladdin Biochemical Technology Co., Ltd. (Shanghai, China). The other chemical reagents, such as C_2_H_4_O_2_, H_2_O_2_ (30%), CH_3_CH_2_OH, and CH_3_COCH_3_, were purchased from Sinopharm Chemical Reagent Co., Ltd. (Shanghai, China). All chemical reagents are analytically pure. Deionized water (18.2 MΩ cm) was used throughout the experiments.

Stone processing wastewater was used as a corrosive medium, which was provided by a factory in Hubei (China). Several parameters of stone processing wastewater and the ionic composition of the solution can be referred to in our previous study [9,10].

### 4.2. Synthesis

#### 4.2.1. Synthesis of the Water-Soluble Chitosan

First, chitosan (2 g) and 2 wt% acetic acid (60 mL) were dissolved and mixed in a beaker containing 250 mL of water. Then, 60 mL of 30% H_2_O_2_ was added into the beaker and reacted for 4 h at 50 °C, and then the mixture was cooled at room temperature for 12 h. Second, 60~80 mL of anhydrous ethanol was added into the beaker, followed by stirring for 1 h, and then the mixture was cooled and let to stand for 12 h at 4 °C. Finally, the mixture was centrifuged at 6000 rmp for 4 min in a centrifuge. The precipitate was heated in a 60 °C oven to evaporate ethanol, thus grounding it to obtain a pale yellow powder, which was water-soluble chitosan (WSC, 89.5% yield).

#### 4.2.2. Synthesis of Water-Soluble Chitosan from Quaternary Ammonium Salts (QWSC)

First, 1 g of WSC was added to a 250 mL round-bottom flask containing 100mL of water. After the WSC was completely dissolved, 3 g of 2,3-epoxypropyltrimethylammonium chloride was added and reacted at 75 °C for 48 h. Then, the mixture was cooled at room temperature. Second, 30 mL of anhydrous ethanol was added into the beaker, followed by stirring for 1h, and then the mixture was cooled and let to stand for 12 h at 4 °C. Finally, the mixture was filtered, and the obtained precipitate was dried in a 60 °C oven and grounded into a fine powder. The prepared product was quaternary ammonium salt water-soluble chitosan (QWSC, 81.2% yield).

### 4.3. Methods

#### 4.3.1. Weight Loss Method

A3 carbon steel coupons (50 mm × 25 mm × 2 mm) were burnished using silicon carbide paper and sonicated in absolute ethanol and deionized water for 5 min, respectively, and dried with a clean tissue. Then, they were weighed using an electronic balance and dried with a blowing machine. Finally, the materials were completely dipped in 1 L of corrosive media without and with QWSC inhibitor. The coupons were immersed under specific conditions of QWSC inhibitor concentrations (20, 40, 60, and 80 mg·L^−1^) and at the speed of 250 rpm for 7 d. After testing, the corrosion products were removed through deionized water, absolute ethanol, and ultrasonic cleaner and dried with tissue. After being dried, all the coupons were weighed again and the change in their weight was recorded. The rates of corrosion (C_R,_ g·cm^−2^ h^−1^) and corrosion inhibition efficiency ($IE_{w}$) were calculated using Equations (3) and (4) [12]:

(3) $$C_{R}=\Delta W/(\mathrm{At})$$

where ΔW is the weight differences (g) of the corresponding coupons before and after the experiment, A is the superficial area (cm^2^), and t is the soaking time (h) in the corrosion medium.

(4) $${\mathrm{IE}}_{w}\%=(C_{R}^{0}-C_{R}){/C}_{R_{\;}}^{0}\times 100$$

where C^0^_R_ and C_R_ are the corrosion rate (g·cm^−2^ h^−1^) of the samples in uninhibited and inhibited solutions, respectively.

#### 4.3.2. Electrochemical Test Method

Electrochemical tests were conducted using a CHI604E electrochemical analyzer in a conventional three-electrode system, which was used for open-circuit potential (OCP), potentiodynamic polarization curves measurements, and electrochemical impedance spectroscopy (EIS). In the three-electrode system, the burnished coupon samples with an exposed area of 1 cm^2^ were used as the working electrode, a platinum electrode was utilized as the auxiliary electrode, and Ag/AgCl served as the reference electrode. Electrochemical experiments were performed in stone processing wastewater corrosive media without the QWSC inhibitor (blank) and with concentrations of the QWSC inhibitor ranging from 20 to 80 mg·L^−1^. Potentiodynamic polarization curves were recorded in the potential range from −700 to 300 mV versus OCP at the scan rate of 10 mV·s^−1^. EIS tests were conducted under OCP with an amplitude of 10 mV by changing the frequency from 100 kHz to 0.01 Hz [9,10]. EIS spectra were fitted utilizing ZSimpWin 3.6 software. The inhibition efficiency in this case was calculated according to the following Equation (5) [10]:

(5) $$\mathrm{IE}\%={(i}_{\mathrm{corr}}^{0}-i_{\mathrm{corr}}{)/i}_{\mathrm{corr}}^{0}\times 100$$

where i^0^_corr_ and i_corr_ are corrosion current densities in the absence and presence of the QWSC inhibitor, respectively.

#### 4.3.3. Quantum Chemical Calculations Method

Quantum chemical calculations were performed using Gaussian 09W software based on Density Functional Theory (DFT) and the B3LYPF method to calculate the optimal geometrical distribution of the single corrosion inhibitor molecules under study and various quantum chemical parameters, including highest occupied molecular orbital energy (E_HOMO_), lowest unoccupied molecular orbital energy (E_LUMO_), energy gap value (ΔE), dipole moment (µ), electronegativity (χ), hardness (η), softness (σ), electrophilicity power (ω), nucleophilicity (ɛ), and the fractional transfer of electrons from the corrosion inhibitor molecule to the metal surface (ΔN). The various performance indicators of the quantum chemical reaction were calculated as follows [33,34].

(6) $$\Delta E=E_{\mathrm{LUMO}}-E_{\mathrm{HOMO}}$$

(7) $$\chi =-\mu =-\frac{E_{\mathrm{LUMO}}+E_{\mathrm{HOMO}}}{2}$$

(8) $$\eta =\frac{\Delta E}{2}$$

(9) $$\sigma =\frac{1}{\eta }$$

(10) $$\omega =\frac{\chi^{2}}{2\eta }$$

(11) $$\varepsilon =\frac{1}{\omega }$$

(12) $$\Delta N=\frac{\chi_{\mathrm{Fe}}-\chi }{{2(\eta }_{\mathrm{Fe}}+\eta )}$$

where χ_Fe_ and χ represent the electronegativity of the iron matrix and the corrosion inhibitor, respectively; η_Fe_ and η represent the chemical hardness of the iron matrix and the corrosion inhibitor, respectively. The theoretical value of χ_Fe_ = 7 eV was used, assuming that in the iron matrix, E_HOMO_ = E_LUMO_; therefore, η_Fe_ = 0. A positive value of the calculated ΔN indicates that the molecule behaves as an electron acceptor during adsorption, and a negative value of ΔN indicates that the molecule is an electron donor, where the corrosion inhibition efficiency of the corrosion inhibitor increases with the electron supplying capacity if ΔN < 3.6 [35,36].

### 4.4. Surface Analysis

The carbon steel coupons were immersed in the solution for 6 h with and without QWSC corrosion inhibitors. After that, they underwent a rinse with deionized water and drying, and then the morphology of the corrosive material surface was observed using a scanning electron microscope (SEM, TESCAN MIRA LMS, Tescan, Brno, Czech Republic) with an accelerating voltage of 15 kV. An atomic force microscope (AFM, Bruker Dimension Icon, Billerica, MA, USA) was adopted to illustrate the roughness of the corrosive material surfaces that were dipped in stone processing wastewater without and with the QWSC inhibitor for 7 d. For the X-ray photoelectron spectroscopy analysis (XPS, Thermo Scientific Nexsa, Madison, WI, USA), Al Kα (1486.6 eV) was adopted as the excitation source. For the calibration, all of the spectra were referenced to the binding energy of C 1s (284.8 eV). The hydrophilicity and hydrophobicity of the corrosive material surface before and after the QWSC inhibitor handling were analyzed using a contact angle instrument (Dataphysics, OCA 25, Filderstadt, Germany).

## 5. Conclusions

In this work, the QWSC inhibitors were synthesized and evaluated as corrosion inhibitors for A3 carbon steel in stone processing wastewater. Electrochemical techniques, a surface analysis, and quantum chemical calculations were employed to elucidate the corrosion inhibition performance. More importantly, the corrosion inhibition mechanism of corrosion inhibitors was revealed. The main results are as follows:

(1) The QWSC inhibitor exhibited slightly superior performance, reaching a maximum efficiency of 59.51% when its addition amount was 60 mg·L^−1^. Electrochemical research showed that QWSC was a mixed-type corrosion inhibitor.

(2) Surface analysis techniques (SEM, AFM, and XPS) and contact angle measurements indicated that the corrosion inhibitor formed an adsorption film on the surface of carbon steel, which generated a corrosion inhibition effect.

(3) Quantum chemical calculations and a corrosion inhibition mechanism revealed that QWSC can also be attached to carbon steel surface via chemisorption through the adsorption center and the formation of coordination bonds with the empty orbitals of the Fe atoms of carbon steel, providing an effective barrier against corrosive environment.

## Figures and Tables

**Table 1:** The polarization curve parameters of A3 carbon steel in stone processing wastewater with different concentrations of QWSC inhibitor.

C mg·L^−1^	E_corr_ mV	*i*_corr_ µA·cm^−2^	*θ*
0	−944	0.448	—
20	−989	0.387	0.136
40	−994	0.265	0.409
60	−994	0.180	0.598
80	−994	0.215	0.520

**Table 2:** Fitting results of EIS parameters.

c mg·L^−1^	R_s_ Ω·cm^2^	R_ct_ Ω·cm^2^	*IE_EIS_*%
0	169.5	675.7	-
20	191.3	757.9	10.85
40	193.2	1052	35.77
60	193.0	1654	59.15
80	189.8	1568	56.91

**Table 3:** Calculated quantum chemical parameters of the compounds.

Inhibitors	WSC	QWSC
E_HOMO_	−6.6439	−5.8540
E_LUMO_	0.0521	0.0229
ΔE	6.6961	5.8769
χ	3.2959	2.9156
ω	1.6222	1.4464
ɛ	0.6164	0.6914
η	3.3481	2.9385
σ	0.2987	0.3403
µ	−3.2959	−2.9156
ΔN	0.5532	0.6950

## Data Availability

The data presented in this study are available upon request from the corresponding author.
